# Genetic Influences on Smoking Behavior and Nicotine Dependence: a Review

**DOI:** 10.2188/jea.13.183

**Published:** 2007-11-30

**Authors:** Kouichi Yoshimasu, Chikako Kiyohara

**Affiliations:** 1 Department of Neuropsychiatry, Graduate School of Medical Sciences, Kyushu University.; 2 Department of Preventive Medicine, Graduate School of Medical Sciences, Kyushu University.

**Keywords:** nicotine, tobacco use disorder, polymorphism (genetics), mental disorders, smoking

## Abstract

Molecular epidemiologic studies suggest genetic factors in the etiology of smoking behavior. Dopamine receptor genes, transporter genes (serotonin and dopamine), and other genes related to metabolism of nicotine are plausible functional candidate genes. Research examining the role of allelic variation in those genes is being actively pursued with respect to nicotine dependence as well as personality characteristics and mental disorders. Some of these genes were reported to be connected with schizophrenia. Although nicotine dependence itself is one of the mental disorders according to the fourth edition of Diagnostic and Statistical Manual of Mental Disorders and the World Health Organization’s International Statistical Classification of Diseases and Related Health Problems-10 nomenclature, the high comorbidity between nicotine dependence and other mental disorders such as schizophrenia or affective disorders has been noted. Therefore, the relationship between those mental disorders and tobacco addiction should be cleared up considering the interactive effect of genetic and environmental factors.

In spite of the great amount of anti-smoking education,^1^ there is still 29% of all people in the United States who continue to smoke^2^ and 47.3% in Japanese men^3^ although the number has decreased gradually. The psychoactive component of tobacco is nicotine which affects the central nervous system. Nicotine is believed to produce its positive reinforcing and addictive properties by activating the dopaminergic pathway projecting from the ventral tegmental area to the cerebral cortex and the limbic system.

The fourth edition of Diagnostic and Statistical Manual of Mental Disorders (DSM-IV) lists three nicotine-related disorders; nicotine dependence, nicotine withdrawal, and nicotine-related disorder not otherwise specified.^2^ Several recent studies have suggested a genetic predisposition toward nicotine dependence, and several specific genes related to nicotine dependence have been identified. Major studies regarding the candidate genes and tobacco addiction in the second half of the 1990’s were shown in Table 1.

**Table 1.** Tobacco addiction and its candidate genes

Researcher (year)	Ethnicity and prevalence of smokers	Association between smoking behavior and allele (genotype)
**1) *DRD2 Taq* IA polymorphism**		
Noble, *et al.* (1994)^38^	Non-Hispanic Caucasians (n = 354)	Risk of genotypes A1/A1 and A1/A2 combined;
	Current smokers, 16%	Current-smokers vs. nonsmokers; OR = 2.15 (1.11-4.15)
	Past smokers, 32.5%	Past smokers vs. non-smokers; OR = 1.71 (1.01-2.89)
	Non-smokers, 51.4%	
Comings, *et al.* (1996)^51^	Non-Hispanic Caucasians (n = 1026)	Prevalence of A1 allele;
	Smokers, 30.4%	Smokers (48.7%) and general populations (25.9%)
	Controls (general population, prevalence of smokers was not known), 69.6 %	A1 allele was inversely associated with the age of onset of smoking and the maximum duration of time the smokers had been able to quit smoking.
Singleton, *et al.* (1998)^52^	Caucasians (n = 221)	Risk of genotypes A1/A1 and A1/A2 combined;
	Smokers, 47.1%	Smokers vs. non-smokers; OR = 0.60 (0.33-1.07)
	Non-smokers, 52.9%	
Spitz, *et* al. (1998)^48^	Non-Hispanic Caucasians (n = 126)	Risk of genotypes A1/A1 and A1/A2 combined;
	Ever smokers, 89.7%	Ever smokers vs. never smokers; OR = 2.0 (0.48-11.9)
	Never smokers, 10.3%	
Lerman, *et al.* (1999)^78^	Americans (n = 522, whites were 85% and 15% of blacks)	Risk of genotypes A1/A1 and A1/A2 combined;
		Smokers vs. non-smokers; OR = 1.2 (0.83-1.75)
	Smokers, 55.4%	
	Non-smokers, 44.6%	
Yoshida, *et al.* (2001)^50^	Japanese (n = 332)	Risk of genotypes A1/A1 and A1/A2 combined;
	Current smokers, 23.2%	Current-smokers vs. non-smokers; OR = 0.44 (0.25-0.79)
	Past smokers, 17.2%	Past smokers vs. non-smokers; OR = 0.29 (0.15-0.55)
	Non-smokers, 59.6%	

**2) *DRD2 Taq* IB polymorphism**		
Spitz, *et al.* (1998)^48^	Non-Hispanic Caucasians (n = 126)	Prevalence of genotypes B1/B1 and B1/B2 combined;
	Ever smokers, 89.7%	30.9% in ever smokers and 0% in never smokers
	Never smokers, 10.3%	

**3) *DRD4* VNTR polymorphism**		
Shields, *et al.* (1998)^56^	Caucasians (n = 403)	Risk of genotypes L/L and L/S combined;
	Smokers, 58.3%	Smokers vs. nonsmokers, OR = 1.04 (0.63-1.79)
	Nonsmokers, 41.7%	
Shields, *et al.* (1998)^56^	African-Americans (n = 72)	Risk of genotypes L/L and L/S combined;
	Smokers, 63.9%	Smokers vs. nonsmokers, OR = 8.04 (1.60-76.77)
	Nonsmokers, 36.1%	

**4) *5-HTT* gene**		
Ishikawa, *et al.* (1999)^73^	Japanese (n = 387)	Risk of genotypes L/L and L/S combined;
	Smokers, 52.2%	Smokers vs. nonsmokers, OR = 1.9 (1.2-2.9)
	Nonsmokers, 47.8%	

**5) *SLC6A3* gene (allele 9 polymorphism)**		
Lerman, *et al.* (1999)^78^	Americans (n = 522, 85% was whites and 15% was blacks)	Risk of genotypes 9/9 and 9/wt combined;
		Smokers vs. non-smokers; OR = 0.69 (0.49-1.00)
	Smokers (n = 289)	Individuals with SLC6A3-9 genotypes were significantly less likely to be smokers especially if they also had DRD2-A2 genotypes.
	Non-smokers (n = 233)	
Sabol, *et al.* (1999)^79^	Mixed populations(n =1107, 84% was non-Hispanic Caucasian)	Risk of genotypes 9/9 and 9/wt combined;
		Current and past-smokers vs. non-smokers; not significant
	Non-smokers, 53.6%	Current smokers vs. non-smokers; OR = 1.49 (1.05-2.11)
	Past-smokers, 20.9%	
	Current-smokers, 25.6%	
Jorm, *et al.* (2000)^80^	Caucasians (n = 861)	Risk of genotypes 9/9 and 9/wt combined;
	Current smokers, 52.4%	Current-smokers vs. non-smokers, OR = 0.98 (0.66-1.32)
	Past-smokers, 30.0%	Past smokers vs. non-smokers; OR = 1.09 (0.77-1.52)
	Never-smokers, 24.5%	

**6) *CYP2A6* gene**		
Pianezza, *et al.* (1998)^91^	Ethnicity, not shown (n = 428)	Risk of null alleles;
	Tobacco-dependants, 57.0%	Tobacco dependants vs. never-dependants, OR = 1.74 (1.02-2.94)
	Never-tobacco-dependants, 43.0%	

**7) *CYP2D6* gene**		
Cholerton, *et al.* (1996)^92^	Caucasians (n = 204)	Risk of non-wild type alleles;
	Smokers, 49.0%	Smokers vs. nonsmokers, OR = 1.21 (0.67-2.18)
	Non-smokers, 51.0%	

VNTR: Variable number of tandem repeat

OR: odds ratio

Wt: wild-type

95% confidence intervals in parentheses.

In addition, patients with mental illness, such as schizophrenia, manic depression, posttraumatic stress disorder (PTSD), and attention-deficit hyperactivity disorder (ADHD), have a higher incidence of smoking than the general population and are the major consumers of tobacco products.^4^ Especially, it is considered that almost 90% of outpatients with schizophrenia smoke.^2^ The recent development of molecular biology regarding genetic influences on schizophrenia or other major mental disorders has provided some evidence for the involvement of a number of specific genes although the results are not necessarily consistent.^5^^,^^6^

Interestingly, some genes which are considered to be associated with these mental disorders overlap with those which are connected with nicotine dependence (e.g. the D2 dopamine receptor (*DRD*2) gene).

Genetic difference is known for substances in tobacco smoke absorption, their metabolism and for their interactions with receptors. In this paper, we discuss the relationship between smoking behavior and mental disorders, with special emphasis on dopamine receptor genes, transporter genes (serotonin and dopamine), and other genes related to metabolism of nicotine.

## Definition of Nicotine Dependence

The widely used generic criteria for drug dependence are the DSM-IV criteria and the World Health Organization International Statistical Classification of Diseases and Related Health Problems (ICD)-10 criteria. The DSM-IV criteria are generally much more detailed than ICD-10, but share common concepts of difficulty in controlling the use of the drug, of giving priority to drug use over other important obligations, to continued use of the drug in the knowledge of harmful consequences, and tolerance to the effects of the drug. The criteria are designed to apply generically to substance abuse, but provide a suitable framework for determining the addictive or dependent nature of nicotine and smoking. Nicotine dependence is the most prevalent (20% lifetime prevalence)^7^ and most deadly (50% die from complications)^8^ of the disorders listed in the DSM-IV and ICD-10.

The DSM-IV diagnosis of nicotine dependence requires a minimum of three of seven diagnostic symptoms: tolerance (needing more of the substance to get the same effect or having less effect with repeated use of the same amount of substance), withdrawal (subjective and/or objective changes in the person when the substance is stopped), greater use than intended, persistent desire to quit, great amounts of time spent smoking, activities given up or reduced due to smoking, and continued smoking despite knowledge of having a persistent physical or psychologic problem with the substance. The vast majority of cigarette smokers are daily smokers and, of these, the majority are nicotine-dependent smokers by DSM-IV criteria.^9^

Questionnaire methods have also been used extensively to measure nicotine dependence. Probably the most widely used is the Fagerström Test for Nicotine Dependence (FTND). The FTND, further refined version of the Fagerström Tolerance Questionnaire, assesses nicotine dependence (Appendix).^10^ The total score ranges from 0 to 10 and the average score in representative samples of smokers is usually around 3. A maximum of 10 points can be reached, which would mean severest dependence, a score below 5 is generally considered mild, 5-6 moderate and at least 7 severely dependant. This scale can be used in a clinical setting, using the score of FTND as a guide.

The FTND may provide a stronger measure of physical dependence, while the DSM may tap other domains such as awareness of dependence, behaviors resulting from that awareness, and psychiatric symptomatology. Although the FTND and the DSM may appear to measure different aspects of the tobacco dependence process,^11^ the term ‘nicotine dependence’ in this review has been used in case of satisfiying either the diagnostic criteria or the questionnaire.

## Twin Study: The Evidence for Genes Involved in Smoking Behavior

Initial support for a genetic influence on the use of tobacco came from cross-sectional studies in twins and showed a mean heritability (that is, the proportion of phenotypic variation attributed to genetic variation) of cigarette smoking of 0.53 (range, 0.28 - 0.84).^12^^,^^13^

Carmelli et al.^13^ used data from a registry of male twins to examine hereditary influences on specific aspects of smoking behavior such as never smoking, former smoking and quitting. A genetic influence on each of these aspects of smoking behavior was observed. In an initial survey in 1967-1969, concordance was higher among mono- than dizygotic twins for both never and former smoking. Quitting during the subsequent 16-year interval was also more commonly observed among mono- than among dizygotic twins. The concordance rates for smoking initiation were 76% in monozygotic twins and 61% in dizygotic twins while among smokers the liability to nicotine dependence was substantially influenced by genetic factors, with an estimated total heritability of 72%.^14^ Their findings were consistent with prior studies showing substantial heritability for variables that may indirectly reflect nicotine dependence such as smoking persistence,^15^ heavy smoking,^16^ and number of cigarettes consumed.^17^ Although twin studies provided supportive evidence for the role of genes in smoking behavior,^18^^,^^19^ none have provided clues as to specific genes that may be involved.

## Receptor Genes

Postsynaptic dopamine receptors can be classified as D1 class (D1 and D5) or D2 class (D2, D3 and D4).^20^ An intriguing aspect of dopamine receptor function is that, under normal conditions, concomitant stimulation of dopamine receptors of the D1 and D2 classes is required for manifestation of many of the behavioral and electrophysiologic effects of dopamine.^21^^-^^23^ This phenomenon has been referred to as requisite D1/D2 synergism.^23^ There are differences between the DRD2 and DRD4, however such as in dopamine affinity (greater for the DRD4 in the low-affinity receptor state) and in the levels of protein expression.^24^ There also is a difference for the binding of the dopamine agonist clozapine, which is an order of magnitude higher for the DRD4 compared with the DRD2.^25^^-^^28^ Clozapine is used for the treatment of schizophrenia, a disease that has a hypothesized underlying defect in the dopamine reward system.

Evidence for genetic determinants affecting the smoking phenotype has steadily accumulated both from studies of substance abuse in animals and from analysis of the contributions of genetics and personality to substance abuse in humans.^29^^,^^30^ Two recent linkage studies in humans^31^^,^^32^ have indicated regions of the genome in which loci affecting nicotine dependence and ever smoking may be found with further work. However, an appreciation of the neurotransmitter-related mechanisms involved in reward circuits in the human brain had suggested many candidate loci potentially associated with smoking behavior.^33^

The human *DRD2* gene has been mapped to a locus on human chromosome 11q23 and several polymorphisms of the gene have been reported.^34^ Interindividual differences in the structure and expression of this gene results in alternation in dopamine availability to tobacco smoking. A polymorphism, an uncommon *Taq* I A restriction fragment length polymorphism (RFLP), (A1 or A2 allele) located in the 3′-flanking region of the *DRD2* gene.^35^ Next, Hauge et al.^36^ found that the more 5′ *Taq* I B RFLP (Bl or B2 allele) is closer to the regulatory and structural coding regions of the gene. The A1 allele has been shown to be associated with low DRD2 density in human brain, compared with the A2 allele.^37^ It has been hypothesized that such persons who have a reduced DRD2 density have a deficit in their reward system and experience an enhanced reward when exposed to dopaminergic agent, thereby making them more prone to nicotine dependence.^38^^,^^39^ Therefore, the A1 allele (or low DRD2 density) has been reported to be significantly associated with increased risk of alcoholism,^40^^,^^41^ abuse of various drugs^42^^-^^44^ and obesity.^45^^,^^46^

Bierut et al.^47^ reported that genetic variation in the *DRD2* locus did not play a significant role in increasing the risk of smoking although the dopamine system may play a significant role in nicotine dependence. Recently, Spitz et al.^48^ found that the *Taq* I B RFLP was a better maker for smoking behavior than *Taq* I A RFLP. There is substantial linkage disequilibrium between the A1 and B1 allelic variants.^49^^,^^50^ Three studies have shown a relationship between either *Taq* I A or *Taq* I B RFLP and genetic predisposition to smoking behavior^38^^,^^48^^,^^51^ while no association was confirmed in another study.^52^ Surprisingly, the A2 allele was associated with smoking behavior among Japanese.^50^ The *DRD2* work is quite controversial. A considerable variation in the prevalence of the A1 allele (0.09-0.75) has been reported by Barr and Kidd.^53^ Several studies have also reported that various populations have the A1 allele frequencies of 0.12-0.24 among Caucasian,^48^ 0.36 among Japanese,^50^ 0.37 among African-American^54^ and 0.42 among Mexican-American.^54^ The prevalence of the A1 allele is higher among the colored than Caucasian. Ethnic variation of this gene may explain why those studies have been inconsistent. A low frequency of the variant allele may fail to establish the association due to low statistical power.

There is an imperfect 48-bp variable number of tandem repeat (VNTR) polymorphism involving 18 amino acids in the third exon of the *DRD4*. The relationship of the *DRD4* exon3 VNTR polymorphism to smoking behavior has received little attention to date. George et al.^55^ found that there was an extremely high incidence (70-90%) of cigarette smoking among alcoholics with the repeated *DRD4* polymorphism. An association between the *DRD4* gene exon 3 polymorphism and smoking was also reported in African-Americans.^56^ Further studies are needed to determine the role of the D4 receptor polymorphism.

The DRD5 binds dopamine 10 times more avidly than the DRD1.^57^ Genetic linkage and association data suggest that the *DRD5* may be positional candidate gene. Vanyukov et al.^58^ reported a significant association between the microsatellite polymorphism D5 (CT/GT/GA9)_n_ located about 55 kb from the *DRD5* gene with a heterogeneous set of psychoactive substance dependencies. However, *DRD5* was not substantially related to smoking initiation and progression to nicotine dependence.^59^

Nicotine is considered the most addictive component in tobacco product.^60^ Nicotine, via binding to neuronal nicotinic receptors, enhances dopamine release and neurotransmission in the mesolimbic dopamine system. Neuronal nicotine acetylcholine receptor (*nAChR*) family is believed to be most relevant to nicotine dependence and consists of 12 distinct genes encoding nine *α*-subunits and three *β*-subunits. Dopaminergic neurons express *α*3, *α*4, *α*5, *α*6, *α*7, *β*2 and *β*3 subunits.^61^ The identification and characterization of polymorphisms in the *α*4 subunit of the *nAChR* is an area of active research.^62^^-^^64^ Although several polymorphisms of this gene have been reported, there is no study on smoking behavior and those polymorphisms. No significant associations of the *nAChR β*2 to smoking behavior or level of nicotine dependence were found.^65^ Although the role of *α*6 subunit in the etiology of nicotine dependence remains unknown, this subunit was implicated in the stimulation effect of nicotine on habituated locomotion.^66^ *α*6 subunit was suggested to be an useful tool to understand the mechanisms of nicotine dependence in mice.^67^ On the whole, *nAChR* subunits have been performed on smoking have been negative. Future research in this field might focus on the establishment of new polymorphic sites in those subunits, with importance for function. Functionally important polymorphic alleles involved in smoking behavior might provide valuable tools for the understanding of nicotine dependence.

## Transporter Genes

The serotoninergic system may be implicated in habitual smoking because nicotine increases brain serotonin secretion and nicotine withdrawal had the opposite effect.^68^^,^^69^ The serotonin transporter (5-HTT) is a plausible candidate for smoking predisposition because a polymorphism in the 5′-flanking region of this gene is associated with its transcriptional efficacy.^70^^,^^71^ There are two common alleles, a 44-bp insertion (L allele) or deletion (S allele). The S allele is associated with decreased transcriptional activity compared with the L allele. Serotonin transporter activity has been shown to decrease *in vivo* in the S/S genotype in comparison to activity in the L/S + L/L genotypes. Because the selective serotonin reuptake inhibitor fluoxetine antagonizes the ability of nicotine to evoke hyppocampal noradrenaline release *in vitro*,^72^ it is expected that the S allele would protect against habitual smoking and/or promote successful smoking cessation. Ishikawa et al.^73^ suggested that individuals with the S/S genotype were less inclined to smoke and/or can more easily stop smoking than others. Smoking behavior was influenced by an interaction between neuroticism and the *5-HTT* genotype.^74^^,^^75^

Dopamine transporter (SLC6A3), determinable with a high affinity radioligand [^123^I]-2*β*-carnometoxy-3*β* (4-iodophenyl) tropane, is relatively stable and, thus, a good indicator for the dopaminergic system.^76^^,^^77^ The *SLC6A3* gene has been shown to influence smoking behavior.^78^^,^^79^ However, these observations were not replicated in another subsequent study using non-volunteer white community sample.^80^ These candidate genes studies need to be repeated in larger sample.

## Genes of Nicotine Metabolizing Enzymes

Considerable inter-individual variability exists in the metabolism of nicotine.^81^ Nicotine is absorbed quickly (in seconds) throughout the body on initial dosing^82^^,^^83^ and then is eliminated with a half-life of 2-3 hours.^84^ Nicotine is metabolized principally (approximately 80 %) to cotinine by cytochrome P450 (CYP) 2A6,^85^^-^^87^ which is also responsible for much of the metabolism of cotinine.^88^ CYP2A6 activity varies approximately 50-fold and the basis for constitutive differences in activity has been associated with variant *CYP2A6* alleles encoding inactive enzyme.^89^^-^^91^ A statistically significantly reduced frequency of two *CYP2A6* null alleles in nicotine-dependent smoking-patients versus never nicotine-dependent individuals and a statistically significant inverse association with the number of cigarettes smoked per week have been reported.^91^ After the initial report, there have been several non-replications, and the suggestions that the original results were technically in error producing much higher rates of the null allele than exist in reality.

Nicotine is in part metabolized by CYP2D6.^92^ Phenotyping analyses have indicated that the ratio between the parent compound debrisoquine and the primary metabolite 4-hydroxyde-brisoquine varies 10,000-fold in the Caucasian population.^93^ Some studies suggest that personality characteristics may differ by *CYP2D6* genotype and/or the phenotype and it has been hypothesized that an endogenous neuroactive substance involved in dopamine neurotransmission may be a substrate of CYP2D6 in brain.^94^^-^^96^ Furthermore, more than 30 commonly prescribed drugs such as tricyclic antidepressants, neuroleptic, lipophylic *β*-blockers, morphine related drugs and some antiarrhythmics (class IC) are substrates for CYP2D6.^96^ As the effect of CYP2D6 activity might rise with increasing tobacco consumption, the effect of tobacco smoking on given diseases might rise with increasing CYP2D6 activity.

## Schizophrenia and Smoking Behavior

It has been reported that prevalence of smoking among schizophrenic patients is extraordinary high (70% or greater), and that schizophrenic inpatients are more likely to be smokers than are persons in general population or even other chronic psychiatric inpatients after correcting for other factors.^98^ The reason why so many schizophrenic patients smoke was attributed in part to the ability of nicotine to reduce extraordinary sensitivity to outside sensory stimuli and to increase concentration in such patients.^2^

Nicotine administration also normalizes several sensory-processing deficits seen in this disease. Animal models of sensory deficits have been used to identify specific nicotine receptor subunits that are involved in these brain pathways, indicating that the *α*_7_ nAChR subunit may play a role.^4^ Genetic linkage in schizophrenic families also supports a role for the *α*_7_ subunit with linkage at the *α*_7_ locus on chromosome 15.^4^

To search for evidence of the genetic loci regarding schizophrenia, both candidate gene and genome-wide linkage studies have been in clinical cohorts collected from a variety of populations. Collectively, these works provide some evidence for the involvement of a number of specific genes such as *DRD2*, *DRD3*, *DRD4* gene, or serotonin type 2a receptor (*5HTR-2a*) gene, although these data provide suggestive, but no conclusive evidence for causative genes.^5^ As has been described above, these genes are considered to be associated with smoking behavior. Some of these genes may be also connected with the strong relationship between smoking and schizophrenia.

However, the relationship between smoking and schizophrenia should be explained not only by the genetic factors but also by the environmental factors. The increased early onset of smoking among schizophrenic patients suggests that high smoking rate of schizophrenic patients may increase the prevalence of smoking even among patients who have not yet shown psychotic symptoms.^98^ Therefore, it is hypothesized that smoking among family members with genetic loading for schizophrenia may be a risk factor of giving a rise to the disease. Thus, the interactive effect of environmental factors and genetic factors should be noted for elucidating the strong relationship between schizophrenia and smoking.

## Other Mental Disorders and Smoking Behavior

Smoking behavior also shows strong relationship with other mental disorders, such as manic depression, PTSD and ADHD. Approximately 50% of all psychiatric outpatients and 70% of outpatients with bipolar I disorder smoke.^2^ Bipolar disorder has some phenotypes in common with schizophrenia and also exhibits genetic linkage to the *α_7_* *nAChR* locus.^4^

It has been also reported that PTSD is associated with heavy smoking as well as depression.^99^^,^^100^ However, the pathways including genetic factors responsible for development and perpetuation of smoking in this clinical population have not been identified.

There are several lines of evidence suggesting that the nicotine system may be functionally significant in ADHD.^101^^-^^103^ Kent et al.^104^ found, however, no significant association between variation at the Cfol polymorphism of *α_4_* *nAChR* gene and susceptibility of ADHD.

Comings and Blum^105^ summarized the substance abuse and behavioral disorders related to dopaminergic and opioidergic reward pathways of the brain, and proposed reward deficiency syndrome (RDS) caused by the defects in various combinations of the genes for neurotransmitters such as dopamine, serotonin, or norepinephrine. Tobacco addiction is also considered to be one of the RDS. However, data regarding correlations between smoking prevalence and these polymorphisms are extremely limited. It is likely that additional and convincing genetic polymorphisms of relevance to smoking behavior will be identified in the near future. To determine genetic influences on smoking initiation and/or cessation may allow most effective smoking prevention strategies and reduce the incidence of physical disorders such as lung cancer and ischemic heart disease.

## PERSPECTIVES

Behavioral genetics research has shown that individual differences in smoking behavior are substantially heritable. Twin and familial studies have shown that the genetic factors involved in the initiation and cessation of smoking are partially overlapping but mostly independent. A larger number of candidate genes for smoking behavior have been identified to date.

Of particular interests are studies on smoking behavior and genes that code for those drug-metabolizing enzymes and gene-environmental interactions as well as studies on lung cancer and metabolic polymorphisms.^106^ As shown in Table 1, data regarding correlations between smoking prevalence and polymorphisms in CYP2A6 and CYP2D6 enzymes are limited. The molecular epidemiologic studies of mental disorders that have been carried out to date have rarely looked at a variety of potential gene-environmental interactions, explored associations and interactions with more than one genetic polymorphism^107^ or gender differences.^108^ Because many mental disorders which are represented by schizophrenia are multifactorial diseases, the high coincidence between tobacco addiction and several mental disorders should be reconsidered from this point of view.

In order to avoid some of the problems associated with molecular epidemiologic studies, Todd^109^ has addressed the issue of guidelines for the interpretation of results from those studies of multifactorial diseases. They expect that those studies should contain large sample size, an independent replication followed by initial study, biological plausibility and physiologically meaningful data supporting a functional role of the polymorphism in question. The initial studies showed a substantially increased/decreased risk of developing lung cancer in individuals with specific genotypes.

Rapid advances in high-throughout gene analysis by DNA chip technology will spread up the identification of new mutations in the genes of predisposing smoking. The main task, however, will be to characterize the functional significance of these gene variants in humans. Such efforts are under way, e.g., the Human Genome Project by Human Genome Organisation, which will come to its completion in the near future, or the Environmental Genome Project pursued by the National Institute for Environmental Health Services in the United States.^110^

Although allelic association is powerful method for detecting quantitative genetic influences on complex traits such as cigarette smoking, there are limitations to such studies. First, it is not yet clear whether the association with given polymorphisms are due to modifications of those polymorphisms or to linkage disequilibrium with some other polymorphisms. Second, allelic association can arise as a result of population stratification as well as genuine transmission. Therefore, polymorphisms stated here are some of many different factors, both genetic and environmental, that influence smoking behavior. A fuller understanding of the genetics of cigarette smoking behavior will lead to the clarification of the relationship between those mental disorders and smoking behavior.

## Appendix.

The Fagerström test for nicotine dependence^10^

**Table:**

Questions	Answers	Points
1. How soon after you wake up do you smoke your first cigarette?	Within 5 minutes	3
	6 - 30 minutes	2
	31 - 60 minutes	1
	After 60 minutes	0

2. Do you find it difficult to refrain from smoking in places where it is forbidden e.g. in church, at the library, in cinema, etc.?	Yes	1
	No	0
	

3. Which cigarette would you hate to give up most?	The first one in the morning	1
	All others	0

4. How many cigarettes per day do you smoke?	10 or less	0
	11 - 20	1
	21 - 30	2
	31 or more	3

5. Do you smoke more frequently during the first hours after waking than during the rest of the day?	Yes	1
	No	0

6. Do you smoke if you are so ill that you are in bed most of the day?	Yes	1
	No	0
